# MRP14 (S100A9) Protein Interacts with Alzheimer Beta-Amyloid Peptide and Induces Its Fibrillization

**DOI:** 10.1371/journal.pone.0032953

**Published:** 2012-03-22

**Authors:** Ce Zhang, Yonggang Liu, Jonathan Gilthorpe, Johan R. C. van der Maarel

**Affiliations:** 1 Department of Medical Biochemistry and Biophysics, Umeå University, Umeå, Sweden; 2 Department of Physics, National University of Singapore, Singapore, Singapore; 3 Laboratory of Stem Cell and Tissue Engineering, Chongqing Medical University, Chongqing, China; 4 Umeå Centre for Molecular Medicine (UCMM), Umeå University, Umeå, Sweden; Russian Academy of Sciences, Institute for Biological Instrumentation, Russian Federation

## Abstract

Increasing evidence supports the contribution of local inflammation to the development of Alzheimer's disease (AD) pathology, although the precise mechanisms are not clear. In this study, we demonstrate that the pro-inflammatory protein S100A9 interacts with the A
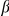
1–40 peptide and promotes the formation of fibrillar 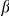-amyloid structures. This interaction also results in reduced S100A9 cytotoxicity by the binding of S100A9 toxic species to A
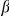
1–40 amyloid structures. These results suggest that secretion of S100A9 during inflammation promotes the formation of amyloid plaques. By acting as a sink for toxic species, plaque formation may be the result of a protective response within the brain of AD patients, in part mediated by S100A9.

## Introduction

Knockdown of S100A9 expression improves cognition function in Alzheimer's disease model mice (Tg2576) and these animals also exhibit reduced amyloid plaque burden [1]. However, a mechanistic link between S100A9 and Alzheimers disease pathology has not been shown. S100A9 protein is a reliable marker of inflammation [2]. Infiltrating macrophages express S100A9 in the event of acute or chronic inflammation [3], [4]. S100A9 and A8 are co-expressed mainly by phagocytes in a variety of inflammatory conditions, including rheumatoid arthritis, allograft rejection, inflammatory bowel, and lung diseases [5], [6]. Inflammatory disorders such as chronic bronchitis, cystic fibrosis, and rheumatoid arthritis are also associated with elevated plasma levels of S100A9 [7], [8].

It has been speculated that inflammation is directly related to Alzheimer's disease. Studies performed in transgenic animals suggest that cerebral amyloid deposition is increased in inflammatory conditions [9]. In these transgenic animals, amyloid plaques do not develop unless inflammation is induced. Heneka et al. reported that in certain conditions related to chronic stress, astrocytes secreting S100 proteins assemble at the site of amyloid plaques, which may serve to prolong neuroinflammation [10]. An additional report shows microglial activation in early stages of AD [11]. These results indicate that early and local inflammatory events contribute to neuronal dysfunction and the formation of amyloid plaques at this and later stages of AD, respectively.

To investigate the relationship between S100A9 and amyloid plaques, we have studied the interaction between the main component of the amyloid plaque (A
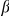
1–40) and S100A9 by exploring the conformational and structural transition of species generated between them by atomic force microscopy (AFM), circular dichorism (CD), polyacrylamide gel electrophoresis (PAGE) and the Thioflovin-T assay (ThT). We observed that, whilst retaining its native structure, S100A9 triggers and promotes the formation of 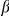-amyloid aggregates. To investigate the effect of S100A9/A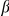 mixtures on cells of neural origin, we employed the WST-1 assay on the neuroblastoma SH-SY5Y cell line. Our results show that freshly dissolved A
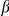
1–40 is not toxic to neural cells, whereas S100A9 shows strong cytotoxicity. The addition of A
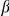
1–40 significantly reduces S100A9 cytotoxicity. Our study provides direct physical evidence that S100A9 triggers and accelerates A
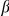
1–40 amyloid formation, which indicates that amyloid plaque formation is related to inflammation and may be the result of a protective response.

## Results

### A
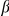
1–40 fibrillization induced by S100A9

A
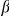
1–40/S100A9 mixtures containing various additions of A
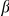
1–40 were examined using atomic force microscopy (AFM). After 1 day (d) of incubation at 37

**Figure 1:**
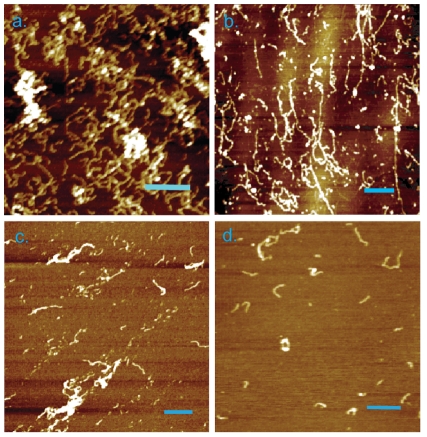
AFM images of freshly dissolved A
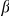
1–40 peptide (0.2 mM) and S100A9 protein (0.02 mM) mixture, incubated at pH 7.4 and 37

C, massive aggregates were produced in the form of round-shaped oligomers, proto-filaments and elongated thick fibrils (Fig. 1). Randomly distributed on mica surface, the round-shaped oligomers had a diameter of 80 nm and a height of 10 nm. The proto-filaments carried a height of 2.7 nm, a width of 50 nm and an average length of 300 nm. They were dimensionally similar to those observed in the A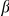 control samples after 3 d of incubation under the same condition (Fig. S1). The thick fibrils were less abundant and had a height of 8 nm, a width of 80 nm and a length in the micrometer range. It is possible that these thick fibrils are formed by the inter-twisting of protofilaments [12]. After incubation for 3 d, there were no obvious transitions in the cross-sectional dimensions of these structures (Fig. 1). The proto-filaments developed into a more extended form with a width of 50 nm and a height of 2 nm. The filaments inter-connected with each other into network-like structures. The co-localization of protofilaments (2 nm in height) and mature fibrils (5 nm in height) supports our supposition that the fibrillar species are initiated from protofilaments.

C for (a) 1 d; and (b) 3 d. S100A9 (0.2 mM) with (c) A
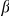
1–40 (0.1 mM) ; (d) A
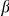
1–40 (0.02 mM) incubated at pH 7.4 and 37

C for 3 d. The scale bars denote 1000 nm for Fig. (a), 1200 nm for (b), 600 nm for (c) and 600 nm for (d).

To identify the origin of massive aggregates, S100A9 was titrated with various concentrations of A
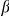
1–40 and incubated at 37

C for 3 d (Fig. 1). As the A
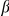
1–40 concentration was reduced, the quantity of aggregates (fibrils and oligomers) decreased dramatically. Using AFM cross-section measurements, we found that these fibrillar structures shared comparable dimensions (2 nm in height and 30 nm in width). Short proto-filaments carrying a similar cross-sectional dimension were observed for concentrations of A
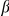
1–40 as low as 0.02 mM. This similarity suggests that these fibrillar aggregates originated from A
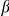
1–40. In conclusion, compared with the amyloid development in A
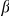
1–40 control samples (Fig. S1: It took 3 d for A
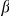
1–40 to develop small quantities of amyloid fibrils with a height of 2 nm), the presence of S100A9 promotes the rate of formation and the quantity of A
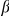
1–40 amyloid species produced.

### Kinetics of amyloid formation

**Figure 2:**
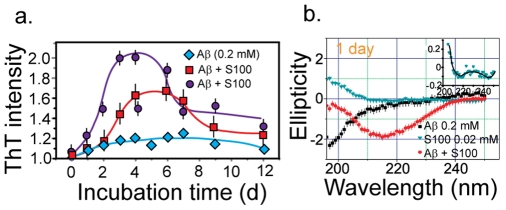
ThT and CD spectra of S100A9/A
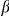
1–40 (0.2 mM) complex. (a) Kinetics of S100A9/A
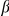
1–40 mixture amyloid formation at pH 7.4 and 37

The kinetics of S100A9/A
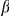
1–40 amyloid formation was monitored using the ThT-binding assay (Fig. 2). The specific interaction of ThT with cross-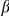-sheet-containing amyloids, leads to an increase in fluorescence emission. The time dependence of amyloid formation depended primarily on the concentrations of A
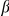
1–40. In contrast to the A
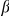
1–40 (0.2 mM) control samples, a sharp increase in ThT intensity was observed with the addition of S100A9 (0.02 mM). After 2 d to 3 d of incubation, the ThT intensity reached its maximum value. A plateau was attained following a decline in the ThT intensity after 7 d to 8 d of incubation. It is possible that the decline in the relative ThT value was induced by the formation of massive aggregates, which is consistent with our AFM observations (Fig. 1). When the concentration of A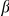 was decreased to 0.1 mM, the relative ThT intensity dropped from 2.0 to 1.6 in spite of the increasing S100A9 concentration (from 0.02 mM to 0.2 mM). The variation in amyloid formation kinetics is consistent with the hypothesis that amyloid formation is largely induced by A
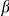
1–40 rather than by S100A9.

C monitored by the thioflavin-T assay. Rhombuses correspond to 0.2 mM A
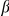
1–40. Squares correspond to 0.2 mM S100A9 and 0.1 mM A
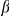
1–40. Circles correspond to 0.02 mM S100A9 and 0.2 mM A
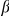
1–40 (b) CD spectra of A
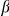
1–40 peptide (0.2 mM), S100A9 (0.02 mM) and their mixture, incubated at pH 7.4 and 37

C for 1 d.

### Transitions in S100A9/A
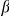
1–40 secondary structures

The transition in secondary structures during the development of S100A9/A
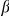
1–40 aggregates was monitored using far UV circular dichorism (CD). The CD spectrum of the control sample containing only A
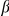
1–40 (0.2 mM) was dominated by random coil structure and characterized by a pronounced negative peak centered at the wavelength below 200 nm after 1 d of incubation at 37

C (Fig. 2) [13]. With the addition of trace amount of S100A9 (0.02 mM) under the same conditions, spectra typical of 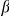-sheet structures (centered at 217 nm) were observed. As there was no obvious secondary-structure transition for the samples containing only S100A9 or A
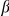
1–40, we conclude that this evolution is caused by the interaction between these two proteins.

To explore the effect of A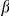 peptide on the secondary structure of S100A9, S100A9 protein was incubated with various concentrations of A
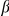
1–40 for 3 d and 7 d. The CD spectra were characterized by two negative peaks centered at 208 nm and 222 nm (Fig. S2). There was little change in the spectra following the addition of A
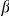
1–40 and incubation at 37

C for 3 d and 7 d. The occurrence of S100A9 amyloid formation was excluded by a comparison with S100A9 samples exposed to extreme conditions (pH 3.0 and continuous shaking at 800 rpm). Under these conditions, S100A9 lost its native structure (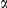 -helix) and took the form of 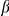-sheet after 3d of incubation (Fig. S4). This transition in the secondary structures of S100A9 is consistent with the downward shift of monomer and dimer band positions in SDS PAGE.

**Figure 3:**
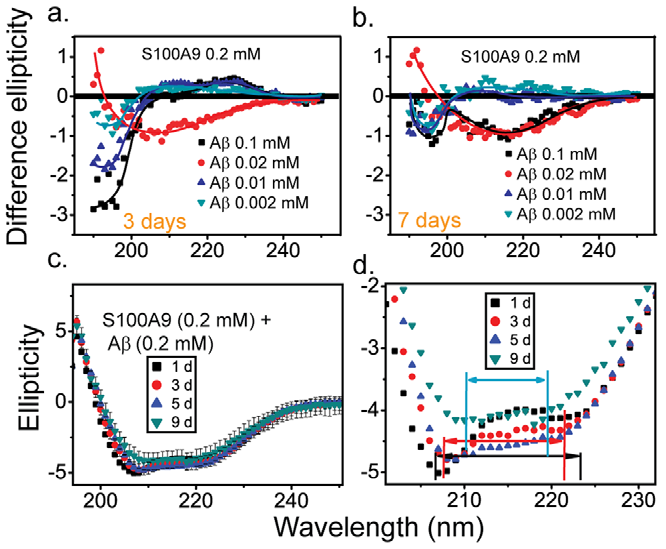
Difference CD spectra of S100A9 (0.2 mM) with the addition of various concentrations A
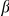
1–40 peptide (0.002 mM to 0.1 mM) incubated at pH 7.4 and 37

The transition in the secondary structures was exaggerated, when the spectra of S100A9 alone were subtracted from the spectra of the mixtures (Fig. 3). Following incubation for 3 d, the difference spectra of the mixtures showed a negative peak with a minimum below 200nm, which is thought to represent random-coil structure. It is worth noting that the absolute values of the ellipticity were proportional to the input concentrations of A
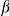
1–40. Prolonged incubation of the mixture with lower concentrations of A
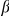
1–40 (0.002 mM and 0.01 mM) for 7 d resulted in a decrease in the absolute value of the ellipticity from 2 to 1. Such transition could represent a shift from random-coil to orderly structures [14]. Unlike other samples, when the A
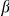
1–40 concentration reached 0.02 mM, the spectra exhibited a shape that is typical for 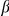-sheet structures. After 7 d of incubation, the negative peak shifted from 206 nm (3 d) to 217 nm. This change denotes the formation of a large quantity of 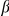-sheet structures [13], [14]. Given that the difference CD spectra were obtained by subtracting the S100A9 signal, we believe the transition to 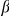-sheet structure is induced by A
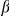
1–40. Considering the lack of transition in the SDS PAGE measurements following the addition of A
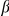
1–40, we believe that the S100A9/A
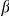
1–40 interaction does not result in any change in S100A9 secondary structures.

C for (a) 3 d; (b) 7 d. The difference CD spectra are obtained by subtracting the ellipticity of the mixture with the ones containing only S100A9 (0.2 mM). (c) Ellipticity of S100A9 (0.2 mM) and A
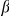
1–40 (0.2 mM) mixture during 9 d of incubation at pH 7.4 and 37

C. (d) Detailed features of panel c.

With prolonged incubation (9 d), minor distinctions were observed in the 200 to 230 nm wavelength range (Fig. 3). The two negative peaks that originate from 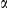 -helix structures showed a tendency to fuse. After incubation for 1 d, the negative peak that centered at 222 nm nearly disappeared and an enhancement in the amplitude of the peak at 208 nm was observed. After 3 d of incubation, there was a decline in the amplitude of the negative peak and a shift in the peak position to 209 nm. The two negative peaks became even less evident when the samples were maintained at 37

C for 9 d. The distance between peaks decreased from 14 nm (208 nm to 222 nm) to 9 nm (210 nm to 219 nm). Taken together, the results from AFM, ThT and CD analyses suggest that S100A9 retains its secondary structures throughout development, and the conformational and structural changes were caused by amyloid formation from the A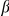 peptide.

### Effect of A
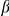
1–40 on S100A9 cytotoxicity

**Figure 4:**
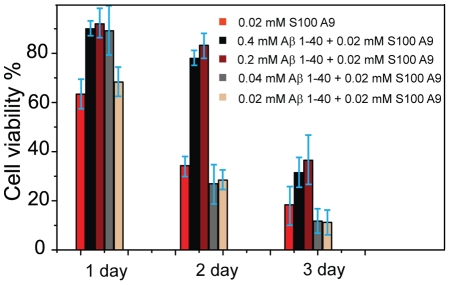
Measurements of NHSY5Y cell line viability by WST1 assay in the presence of 0.02 mM S100A9 and various S100A9/A
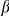
1–40 mixtures. The S100A9 concentration remained 0.02 mM. The concentration of A
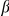
1–40 addition varied from 0.02 mM to 0.4 mM. In control experiments, the cells were incubated in DMEM cell culture buffer alone and the cell viability was equal to 100%.

The effect of S100A9, A
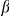
1–40 and their aggregates on the viability of SH-SY5Y neuroblastoma cells was assessed using the WST-1 assay. In viable cells, WST-1 undergoes reduction by mitochondrial dehydrogenases (succinate-tetrazolium reductase system) to soluble formazan, which serves as an indicator of the number of metabolically active cells. The effect of A
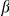
1–40 on S100A9-induced cytotoxicity was investigated by pre-mixing S100A9 with various concentrations of A
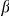
1–40 and incubating the samples with SH-SY5Y neuroblastoma cells for 1 d to 3 d (Fig. 4). During the first day of incubation, at least 0.04 mM A
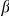
1–40 was required to inhibit the cell cytotoxicity of S100A9 (increasing cell survival to 90% from 70%). After 2 d of incubation, a 50% increase in cell viability (from 30% to 80%) was observed for A
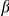
1–40 concentrations exceeding 0.2 mM (0.4 mM and 0.2 mM). Although the cell survival rate for all samples dropped greatly on the third day of incubation, the addition of excessive amounts of A
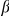
1–40 (0.2 mM and 0.4 mM) increased the cell viability from 10% to 30%. We concluded that the cytotoxicity of S100A9 in SH-SY5Y cells is greatly suppressed by the presence of sufficient amounts of A
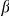
1–40 peptide (more than 0.2 mM A
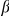
1–40 for 0.02 mM S100A9).

### Characterization of the interaction between A
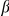
1–40 and S100A9

**Figure 5:**
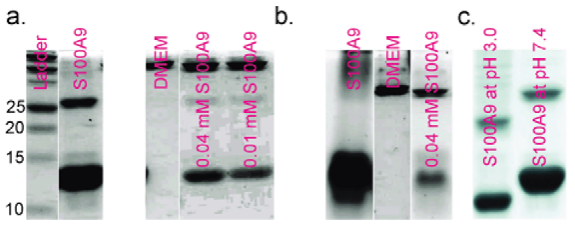
Electrophoresis of S100A9 incubated with NHSY5Y cells. (a) and (c) SDS PAGE; (b) Native PAGE. Panel (a) has the following designations: column 1 corresponds to the ladder; 2 to S100A9 (0.2 mM); 3 to cell culture buffer DMEM; 4 to S100A9 (0.04 mM) with cells in DMEM for 3 d; 5 to S100A9 (0.01 mM) with cells in DMEM. In panel (b), 1 to S100A9 (0.2 mM); 2 to DMEM; 3 to S100A9 (0.04 mM) with cells in DMEM for 3 d. (c) 0.2 mM S100A9 at pH 3.0 and 7.4. The data was obtained by several experiments, and integrated together according to the ladders.

To explore the interaction between S100A9 and A
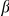
1–40 during incubation with cells, the mixtures and their corresponding controls (S100A9 and A
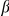
1–40 only) were analyzed using sodium dodecyl sulfate (SDS) and native polyacrylamide gel electrophoresis (PAGE). No significant shifts in protein band positions were observed (Fig. 5). According to the bands generated by the control samples (containing only S100A8, A9, or culture media alone), the uppermost band corresponds to components of the media and the lower two bands represent the S100A9 monomer and homodimer, respectively. Because there was no change in the position of the S100A9 bands, it appears that S100A9 maintained its native structure during incubation with the cells. The structural stability of S100A9 was further verified by comparison with the samples exposed to harsh conditions (pH 3.0 and continuous shaking at 800 rpm). After 1 day of shaking at acidic pH, the band position of S100A9 shifted significantly, indicating the formation of more compact, faster migrating structures. A downward shift in band position corresponds to S100A9 amyloid formation, thereforem the absence of changes could indicate an absence of S100A9 amyloid formation during incubation with the cells.

**Figure 6:**
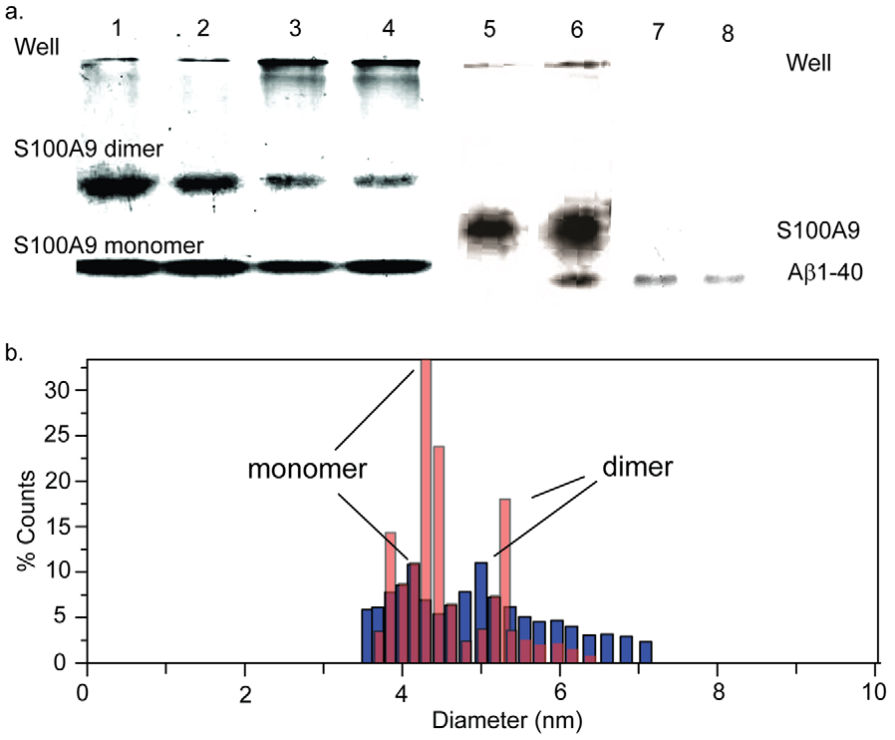
Electrophoresis and gas-phase electrophoretic mobility molecular analysis (GEMMA) of S100A9 and S100A9/A
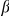
1–40 mixture. (a) SDS PAGE of S100A9 with (1) 0.002 mM; (2) 0.01 mM; (3) 0.02 mM; (4) 0.1 mM A
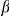
1–40 and native PAGE of (5) 0.2 mM S100A9 with 0.01 mM A
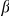
1–40; (6) 0.2 mM S100A9 with 0.1 mM A
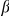
1–40; (7) 0.1 mM A
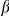
1–40; (8) 0.01 mM A
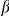
1–40. The final concentration of S100A9 is 0.2 mM. The data was obtained by several experiments, and integrated together according to the ladders. (b) Size distribution of S100A9 monomer and dimer at pH 7.4 and 3.0 examined by gas-phase electrophoretic mobility molecular analysis (GEMMA). The red columns represent S100A9 at pH 7.4. The blue ones represent S100A9 at pH3.0.

The quantities of S100A9 monomer and homodimer were estimated by integrating the intensities of all bands using Image J software. Based on the SDS PAGE data, the freshly dissolved S100A9 contained 74% monomers and 26% homodimers. After incubation with cells for 3 d, the fraction of homodimer dropped to 8.4%, which suggests that the reduction in the quantity of S100A9 dimers is caused by its interaction with cells. A similar decrease in the S100A9 dimer content was observed when S100A9 was mixed with various concentrations of A
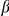
1–40 (Fig. 6). Accompanied by a dramatic increase in the quantity of large aggregates at the top of the gel, a declining intensity in the dimer bands was observed for increasing A
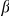
1–40 doses, which indicates the consumption of S100A9 dimers by A
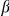
1–40. The fraction of S100A9 dimers was 30% at 0.002 mM A
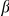
1–40, 18% at 0.01 mM A
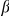
1–40, 14% at 0.02 mM A
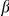
1–40 and 10% with 0.1 mM A
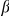
1–40. Native PAGE revealed a band for A
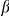
1–40 monomers when the concentration of A
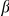
1–40 exceeded 0.1 mM. Given that S100A9 alone contained 74% monomers and 26% homodimers, 0.2 mM S100A9 solution contains 0.15 mM monomers and 0.025 mM dimers. If A
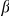
1–40 interacts with the S100A9 monomer, the reaction could consume at least 0.15 mM of A
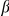
1–40, which is inconsistent with the existence of an A
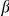
1–40 band in the presence of 0.1 mM A
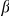
1–40. Conversely, 0.025 mM S100A9 dimers could consume 0.02 mM A
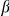
1–40. Considering the decrease in the amount of S100A9 dimers and the absence of an A
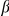
1–40 band for A
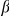
1–40 concentrations below 0.02 mM, we believe that there is a specific interaction between the S100A9 dimer and A
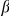
1–40 that does not involve S100A9 monomer. Moreover, there was no additional band for S100A9/A
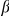
1–40 hetero-dimer or any other visible complexes. Thus, it can be assumed that these proteins are not stabilized in low-molecular-weight structures. It is possible that S100A9 can serve at the A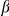 nucleation or fibrillation stages. The rapid formation of A
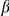
1–40 amyloid structures contributes to the absence of specific stable complexes between S100A9 and A
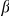
1–40 in low-molecular-weight forms.

## Discussion

The induction of the S100A9 gene was found in the brains of AD patients and AD animal models, Tg2576 and CT-Tg mice [1]. This observation, together with our results, suggests that S100A9 is responsible for the formation of AD amyloid plaques. The detailed molecular mechanism remains however, unknown. Therefore, we focused our research on the role of S100A9 in A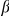 amyloid formation and interaction with neuron origin cells.

The consequences of S100A9/A
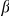
1–40 interaction were investigated using AFM and CD. When S100A9 was mixed with A
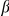
1–40, large quantities of amyloid aggregates (fibrils and oligomers) were formed (Fig. 1). The height of the fibrils was 2 nm, which is similar to the values pertaining to mature A
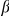
1–40 amyloid fibrils (Fig. S1). Conformational variations with different A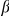 concentrations identified A
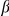
1–40 as the origin of amyloid formation (Fig. 1). In contrast to the lack of transition in the S100A9 secondary structure, A
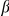
1–40 is responsible for the small changes seen in CD spectra (Fig. 3). The possibility of S100A9 amyloid formation was excluded by comparing S100A9 CD spectra in Fig. 3 with those of S100A9 exposed to harsh conditions (pH 3.0, shaking) (Fig. S3). The formation of S100A9 amyloid structure not only induced the transition to 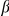-sheet structure accompanied by the formation of small filaments (1 nm in height), but also increased cell viability (Fig. S4). The reduction in S100A9 cytotoxicity induced by the addition of A
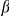
1–40 was further verified by a luminescent cytotoxicity assay of neuron cells (Fig. S5). Sharing the same protective effect against S100A9 cytotoxicity, A
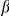
1–40 monomer was more efficient than A
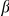
1–40 plaques. The higher cell survival rate with A
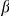
1–40 monomers compared to the ones of the plaques is consistent with our AFM observation that S100A9 interacted with freshly dissolved A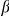 peptides. Possibly, at the same peptide concentration, the pre-formed amyloid plaques decrease A
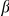
1–40 monomer quantity and S100A9 consumption, which was observed as an increased cytotoxicity in the luminescent cytotoxicity assay. The A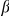/S100A9 interaction was further studied using the peptides A
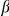
1–16, A
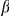
12–14 and A
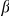
1–42 (Fig. S5). Similar to the case with A
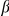
1–40, S100A9 cytotoxicity was greatly reduced upon addition of A
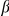
12–14 and A
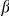
1–42. This was accompanied by the formation of large quantities of amyloid aggregates (Fig. S6). It is worth noting that the A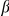/S100A9 interaction did not only inhibit S100A9 cytotoxicity, but also suppressed the toxicity of A
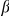
1–42. In contrast, the addition of A
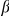
1–16 brought no effect on S100A9 cytotoxicity and no emergence of amyloid fibrillar structures. As the major difference between A
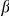
1–16 and other A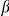 peptides is the absence of a hydrophobic core, we conclude that the hydrophobic core (L17V18F19F20A21) is essential for the S100A9 and A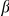 peptide interaction. In summary, S100A9 interacts with A
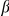
1–40 and induces A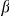 amyloid formation whilst retaining its native structures. This specific interaction reduces the quantity of toxic S100A9 species and thus decreases S100A9-mediated cytotoxicity.

With a constant amount of S100A9 monomers, we observed a great decrease in S100A9 dimer content from 26% to 8% (Fig. 5). Such variation reflects the consumption of S100A9 dimers via interaction with cells, which does not involve S100A9 monomers. Since S100A9, apparently via the activity of the dimmer, induces cell death, the reduction in cytotoxicity induced by the addition of A
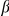
1–40 could be related to its specific interaction with S100A9 dimers. The hypothesis is supported by titrating S100A9 with different dose of A
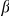
1–40. With increasing concentrations of A
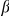
1–40, amount of S100A9 dimer deceased dramatically (Fig. 6). Additionally, more aggregates are formed as indicated by the increasing band intensity corresponding to the wells. Therefore, it is possible that the interaction between S100A9 dimers and A
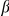
1–40 reduces the number of toxic S100A9 dimers, leading to an increase in cell viability.

**Figure 7:**
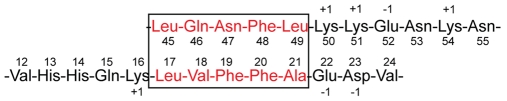
Amino acid sequence alignment of S100A9 in the hinge region and A
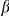
12–24 peptide. The hydrophobic cores of both proteins are marked in red. The charged residues are indicated with their approximate charges. All sequences were obtained from the SWISS-PORT protein database [26].

S100A9 homodimer is shown to be expressed independently during inflammation [15], [16]. It has the ability to induce neutrophil immobilization and bind both Ca and Zn ions [17], [18]. Structurally, the S100A9 monomer consists of 4 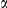-helices (H1,H2,H3 and H4), a long carboxy-terminal tail and a hinge region between H2 and H3. Through hydrophobic interactions, the C- and N-teminus of helices (H1 and H1';H4 and H4') from two monomers make contact with each other, and form an antiparallel-packed hydrophobic core [19]. Because the electron density of 28 residues in C-terminal tail is low and the residues are mostly hydrophilic, it is believed that the residues do not participate in electrostatic and hydrophobic protein-protein interactions [19]. As an exposed part of the intra-molecule hydrophobic cluster, the hinge region is identified as a target-binding site for proteins [20]. The interaction between A
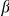
1-40 and S100A9 is possibly initiated by binding to S100A9 hinge region. According to our previous simulation results, the contact of the A
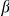
12-24 hydrophobic core (L17V18F19F20A21) leads to the formation of 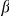-sheet structures, which then act as a seed for further amyloid formation [21]. Besides aggregation, proper stacking (parallel or anti-parallel) is required, which demands a specific residue sequence. The hinge region of S100A9 consists of 11 residues (Leu45-Asn55) (Fig. 7). Similarly to A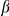 peptides, the hinge region has a hydrophobic core (L44Q45N46F47L48), following which K (50, 51, 54) residues are positively charged and E52 is negatively charged. By electrostatic interaction, E22D23 residues pair with K50K51 residues. Such stacking allows A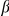 peptide molecules to be aligned in a certain direction. As the anti-parallel stacking is energetically preferred for A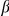 amyloid formation at physiological pH, it is plausible that A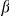 amyloid formation is initiated by binding of A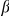 peptide molecules to different sites of S100A9 [20]. The S100A9 homodimer is formed by anti-parallel stacking of two monomers. Accordingly, the binding of A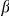 peptides to the hinge regions of two monomers provides the possibility of anti-parallel stacking. This is consistent with our assumption that the S100A9 homodimer interacts with A
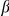
1–40 and our observation of the interactions between S100A9, A
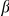
12–24, and A
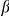
1–42 peptide. Similar to A
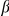
1–40, A
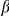
12–24, and A
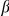
1–42 amyloid formation was greatly promoted by S100A9, which emphasizes the importance of the A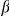 hydrophobic core (Fig. S6). With no hydrophobic core (L17V18F19F20A21), A
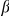
1-16 showed no obvious change upon addition of S100A9 in both AFM and Cytotoxicity experiments.

Overall, the results of this study, combined with those in previous reports, suggest that A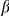 amyloid production is induced by the pro-inflammatory protein S100A9. The A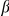-induced reduction of cytotoxicity indicates that the formation of AD amyloid plaques could be a beneficial response to inflammation. It ameliorates the potentially destructive effects of inflammatory molecules such as S100A9. S100A9-induced A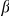 amyloid formation also explains the presence of amyloid plaques in normal aged brain [22], [23]. Further computer simulations will be conducted to elucidate the interaction between S100A9 and A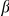 peptides.

## Materials and Methods

### Sample preparation

The proteins used in these experiments were recombinant S100A9 (MRP14) and A
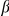
1-40 obtained from Alexo Tech AB(Umea, Sweden); A
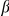
1-42 and A
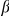
1-16 (Innovagen, Sweden). All experiments were performed with A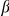 peptide concentrations of 0.02 to 0.2 mM and S100A9 concentrations of 0.02 to 0.2 mM. S100A9 protein solutions were prepared in 10 mM Tris-HCl buffer at pH 7.4, and dialyzed to pH 3.0 in 20 mM Glycine buffer using Slide-A-Lyzer Dialysis Cassettes (Thermo Scientific, USA) at 4

C. We dissolved A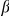 peptide at low temperatures (ice water) following a protocol released previously [24]. The chilled A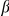 peptide powder was initially dissolved in 10 mM NaOH at a concentration above 1 mg/ml and sonicated in an ice water bath for 1 min. Finally, a trace amount of 1 M 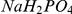 buffer was added to adjust the sample to pH7.4 and 3.0. All other chemicals were purchased from Sigma unless mentioned differently.

#### Amyloid kinetics Assay

A ThT stock solution was made by dissolving 2.5 mM Thioflavin-T (Merck-Schuchardt) in phosphate buffer (10 mM phosphate, 150 mM NaCl, pH 7.4) and filtered before use. This stock solution was diluted 50-fold in the phosphate buffer to generate the working solution. Protein samples of 10 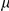
L of protein sample was taken added to 300 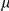
L of the working solution, and was allowed to bind the ThT for 1 min. ThT Fluorescence was measured using a FluoroMax-2 spectrofluorometer (Jobin Yvon/Psex Instruments) with excitation and emission wavelengths of 440 and 48 5nm, respectively, and a slit width of 5 nm. The ThT fluorescence intensities were normalized to the fluorescence of the free dye in solution.

#### CD spectroscopy

CD spectra were recorded with a JASCO J-720 spectropolarimeter equipped with a PTC-343 temperature controller. For each sample, 3 spectra were acquired and averaged in the range of 190 nm to 250 nm, using a 5 nm/min scan speed, and 1 nm resolution. The quartz cells had a 1 mm optical path. The contribution from the buffer was subtracted, and the results are presented in relative ellipticity. The difference ellipticity in Fig. 3 was obtained by subtracting the ellipticity of A
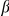
1-40 peptide (0.002 to 0.1 mM) and S100A9 (0.2 mM) complex with the ones from 0.2 mM S100A9.

#### Electrophoresis

SDS and native polyacrylamide gel electrophoresis (PAGE) was carried out on 15% Tris gels. 5 volume of protein sample was mixed with 1 volume of loading buffer (1.6 ml Tris-HCl (1 M), 4 ml SDS (10%), 2 ml glycerol (100%), 1 ml 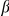-mercaptoethanol, 1.4 ml water and 4 mg Bromophenol blue; SDS and 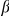-mercaptoethanol are excluded for native-PAGE). The electrophoretic separation was conducted in a Mini Protean II Electrophoresis system (Biorad, USA) with a running buffer (25 mM Tris and 192 mM glycine at pH 8.3; 0.3% SDS was added for SDS PAGE). The standard protein ladder was purchased from BioRad. Coomassie brilliant blue B7920 (Biorad, USA) (0.125 g Coomassie Brilliant Blue R-250 in 200 ml methanol, 35 ml acetic acid and 265 ml water, filtered before use) was used for staining, and the following destaining step was performed by using destaining agent (50% methanol, 10% acitic acid and 40% water) for around 2 hours with gentle agitation.

#### Gas-phase electrophoretic mobility molecular analysis (GEMMA)

The operation procedure of GEMMA instrument was as published previously [25]. S100A9 samples at pH 7.4 and 3.0 were exchanged against 20 mM ammonium acetate buffer (pH 7.4) by centrifugal ultrafiltration in Spin-X Centrifuge Tubes (Sigma-Aldrich, USA) before loading into the capillary tube. The operation current and voltage were approximately 300 nA and 2 kV respectively.

#### Atomic Force Microscopy (AFM)

AFM measurements were obtained with a PicoPlus AFM (Agilent) in tapping mode using a 100 nm scanner in ambient conditions. Acoustically driven cantilevers with etched silicon probes of the TESP model (diameters of 10nm and less) (Digital Instruments) were used. We applied a resonance frequency in the 170 kHz to 190 kHz range, a scan rate of 1 Hz or less, and a resolution of 512 pixels×512 pixels. Height, amplitude and phase data were collected simultaneously in trace and retrace modes to avoid scan artifacts. Amyloid samples were deposited on the surface of freshly cleaved mica (GoodFellow) for 5 min, washed 3 times with 200 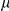
l of DI water and dried in air at room temperature.

#### Cell culture

SH-SY5Y (ATCC CRL-2266) were cultured in Dulbecco's modified Eagle medium supplemented with 10%(v/v) FBS. Mixed cortical neuron cultures were prepared from embryonic day 16.5 rat embryos. Animals used in this study were maintained in strict accordance with the approval granted by Ume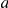 Ethical Committee for Animal Experimentation (Permit Number: A107–08). The cortical hemi-spheres were quickly dissected in ice-cold phosphate saline buffers (PBS, pH 7.2) with 10 mM D-glucose. Cortices from 6–8 embryos were pooled and neurons were isolated using the Papain Dissociation System (Worthington Biochemical Corp). Viable cells were counted following staining with 0.2% (w/v) trypan blue (Gibco/Invitrogen) and plated at a density of 10,000 cells per well (100,000 cells/ml) in a 96-well black-walled imaging plate (BD Falcon). Neurons were cultured for 72 h in Neurobasal medium (Invitrogen) containing 2% (v/v) NS21 supplement (Chen et al. 2008), 0.5 mM L-glutamine, 100 units/ml penicillin and 100 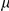
g/ml streptomycin in a humidified incubator (37

C/5% (v/v) 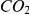).

#### WST1 cell viability assay

To evaluate cell viability, 10 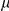
l of WST-1 reagent was added per 100 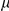
l of cell culture and samples were incubated at 37

C for 4 h. The absorbance was measured with an ELISA plate reader (Labsystem Multiscan RC) at 450 nm. Cell viability was expressed as a percentage of the absorbance in wells containing cells treated with amyloid compared to the control cells.

## Supporting Information

Figure S1
**A
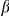
1–40 peptide (0.2 mM) incubated at pH 7.4 and 37

C** for (a) 1 d; (b) 3 d. The scale bars denote 600 nm and 2000 nm, respectively. The insertions show the cross-section profile of the fibrils corresponding to the marked positions. After 1 d incubation, proto-fibrils with a height of 0.5 nm were observed. After 3 d, the surface was dominated by fibrils with a height of 2 nm, which is typical for amyloid mature fibrils. As the fibrils observed upon the addition of S100A9 (Fig. 1) carried a height of 2 nm and the protofibrils are not necessarily related with amyloid formation, it is reasonable to conclude that the presence of S100A9 promotes the rate of formation and the quantity of A
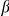
1–40 amyloid species produced.(TIF)

Figure S2
**CD septra** (a) The transition in the secondary structures of (a) S100A9 (0.2 mM); (b) A
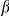
1–40 peptide (0.2 mM). Ellipticity of S100A9 (0.2 mM) with the addition of various concentrations of A
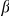
1–40 peptide (0.002 mM to 0.1 mM), incubated at pH 7.4 and 37

C for (c) 3 d; (d) 7 d. The insertions denote the curves of 0.1 mM A
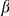
1–40 in panel (c) and (d).(TIF)

Figure S3
**AFM height images of S100A9 (0.2 mM)** (a) incubated at pH 3.0 and 57

C with continuous shaking at 800 rpm for 3 d; (b) incubated at pH 3.0 and 37

C for 3 d; (c) incubated at pH 7.4 and 37

C for 3 d. The scale bars represent 500 nm, 1000 nm and 500 nm, respectively. (d) CD spectra of the corresponding samples shown in AFM: black squares correspond to Fig. (a); blue triangles correspond to Fig. (b); red circles correspond to Fig. (c).(TIF)

Figure S4
**Measurements of NHSY5Y cell line viability by WST1 assay in the presence of** (a): freshly dissolved A
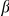
1–40 at pH 7.4. Different colors denote different concentrations of A
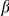
1–40. (b): 0.2 mM A
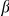
12–24 fibrils, 0.2 mM A
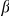
1–40 fibrils and 0.2 mM A
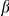
1–40 plaque. A
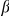
1–40 plaques were formed by incubation at pH 3.0 and 37

C for 3 d. To avoid pH effect, pre-formed A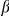 plaques were carefully dialyzed to pH 7.4. A
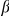
12–24 and A
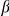
1–40 fibrils were formed by incubation at pH 7.4 and 37

C for over 10 d. (c) The relationship between the kinetics of S100A9 amyloid formation (Relative ThT: left Y axis) at pH 3.0 and the effects of S100A9 (0.02 mM) on NHSY5Y cell viability (WST1 assay: right Y axis). Before mixing with cells, S100A9 was incubated at pH 3.0 and 4

C for up to 70 d. Aliquots were taken for measurements of ThT and cytotoxicity at different time during incubation. The columns in each group correspond to 1 to 3 d of incubation with cells (In day 6 aliquots, S100A9 samples were incubated with cell for up to 2 d). (d) Measurements of NHSY5Y cell line viability by WST1 assay in the presence of S100A9 (0.02 mM) at pH 7.4 and 3.0. Different colors denote 1 d to 3 d of incubation with cells.(TIF)

Figure S5(a) Luminescent cytotoxicity assay of neuron cells in presence of 0.02 mM S100A9 and S100A9/A
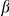
1–40 mixture. S100A9 and the mixture were incubated at 37

C for 3, 6, 12 and 24 h before mixing with cells. A
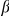
1–40 plaques were formed by incubation at pH 3.0 and 37

C for 3 d. To avoid pH effect, pre-formed A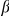 plaques were carefully dialyzed to pH 7.4 before mixing with S100A9. Utilizing cortical neuron cells, the CytoTox-Glo Cytotoxicity assay uses a luminogenic peptide substrate, the AAF-Glo, to measure the activity of dead-cell protease, which is released from cells that have lost membrane integrity. The AAF-Glo substrate cannot cross intact cell membranes and does not generate any appreciable signal from the live-cell population. (b) Measurements of NHSY5Y cell line viability by WST1 assay in the presence of freshly dissolved (1) 0.2 mM A
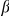
12–24; (2) 0.2 mM A
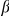
12–24 and 0.02 mM S100A9 mixture; (3) 0.2 mM A
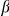
1–16; (4) 0.2 mM A
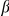
1–16 and 0.02 mM S100A9 mixture; (5) 0.2 mM A
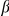
1–42; (6) 0.2 mM A
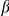
1–42 and 0.02 mM S100A9 mixture.(TIF)

Figure S6
**AFM images of** (a) A
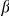
12–24 peptide (0.2 mM) incubated at pH 7.4 and 37

C for 3 d; (b) the mixture of freshly dissolved A
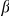
12–24 peptide (0.2 mM) and S100A9 protein (0.02 mM) incubated at pH 7.4 and 37

C for 3 d; (c) A
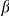
1–42 peptide (0.2 mM) incubated at pH 7.4 and 37

C for 3 d; (d) the mixture of freshly dissolved A
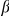
1–42 peptide (0.2 mM) and S100A9 protein (0.02 mM) incubated at pH 7.4 and 37

C for 3 d. In fig. a and b, the scale bars denote 100 nm in the figure, and 1000 nm in the insertion, respectively. The scale bars denote 200 nm in fig. c and 500 nm in fig. d. With the addition of S100A9, large quantities of A
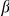
12–24 amyloid fibrils (2 nm in height) were formed, which is similar in size with the mature A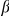 fibrils. In contrast, only protofibrils (0.5 nm in height) were observed with A
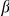
12–24 control samples under the same conditions (panel a). Similarly, A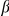 fibrils were only formed in the presence of S100A9 after 3 d incubation at pH 7.4 and 37

C. The average height of the fibrils in fig. d is 2 nm, which is close to the reported value for A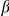 amyloid fibrils.(TIF)
