# Supplementary figures and images for: MRP14 (S100A9) Protein Interacts with Alzheimer Beta-Amyloid Peptide and Induces Its Fibrillization
Source: PLoS One. 2012 Mar 22;7(3):e32953. doi: 10.1371/journal.pone.0032953 (PMC3310843; doi:10.1371/journal.pone.0032953)

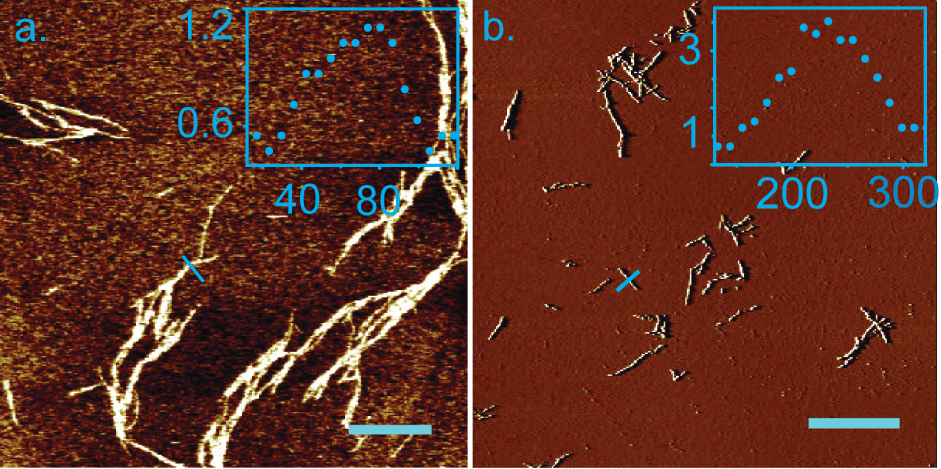

Supplement: Figure S1 — A1–40 peptide (0.2 mM) incubated at pH 7.4 and 37C for (a) 1 d; (b) 3 d. The scale bars denote 600 nm and 2000 nm, respectively. The insertions show the cross-section profile of the fibrils corresponding to the marked positions. After 1 d incubation, proto-fibrils with a height of 0.5 nm were observed. After 3 d, the surface was dominated by fibrils with a height of 2 nm, which is typical for amyloid mature fibrils. As the fibrils observed upon the addition of S100A9 (Fig. 1) carried a height of 2 nm and the protofibrils are not necessarily related with amyloid formation, it is reasonable to conclude that the presence of S100A9 promotes the rate of formation and the quantity of A1–40 amyloid species produced. (TIF) [file pone.0032953.s001.tif]

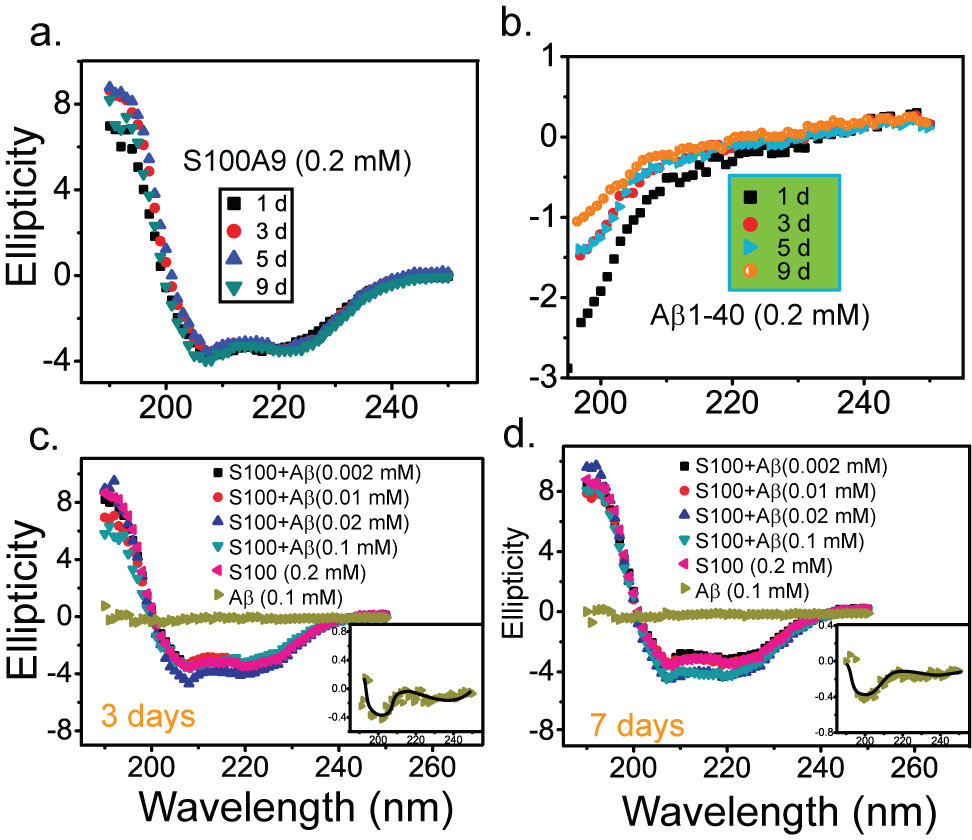

Supplement: Figure S2 — CD septra (a) The transition in the secondary structures of (a) S100A9 (0.2 mM); (b) A1–40 peptide (0.2 mM). Ellipticity of S100A9 (0.2 mM) with the addition of various concentrations of A1–40 peptide (0.002 mM to 0.1 mM), incubated at pH 7.4 and 37C for (c) 3 d; (d) 7 d. The insertions denote the curves of 0.1 mM A1–40 in panel (c) and (d). (TIF) [file pone.0032953.s002.tif]

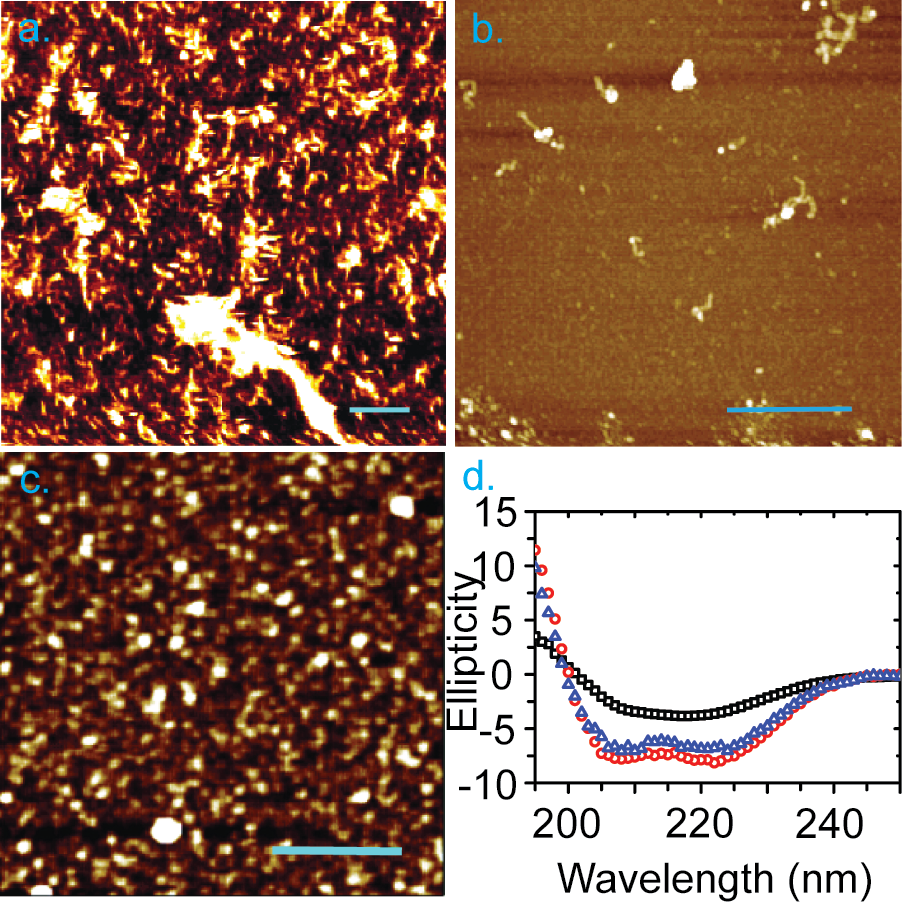

Supplement: Figure S3 — AFM height images of S100A9 (0.2 mM) (a) incubated at pH 3.0 and 57C with continuous shaking at 800 rpm for 3 d; (b) incubated at pH 3.0 and 37C for 3 d; (c) incubated at pH 7.4 and 37C for 3 d. The scale bars represent 500 nm, 1000 nm and 500 nm, respectively. (d) CD spectra of the corresponding samples shown in AFM: black squares correspond to Fig. (a); blue triangles correspond to Fig. (b); red circles correspond to Fig. (c). (TIF) [file pone.0032953.s003.tif]

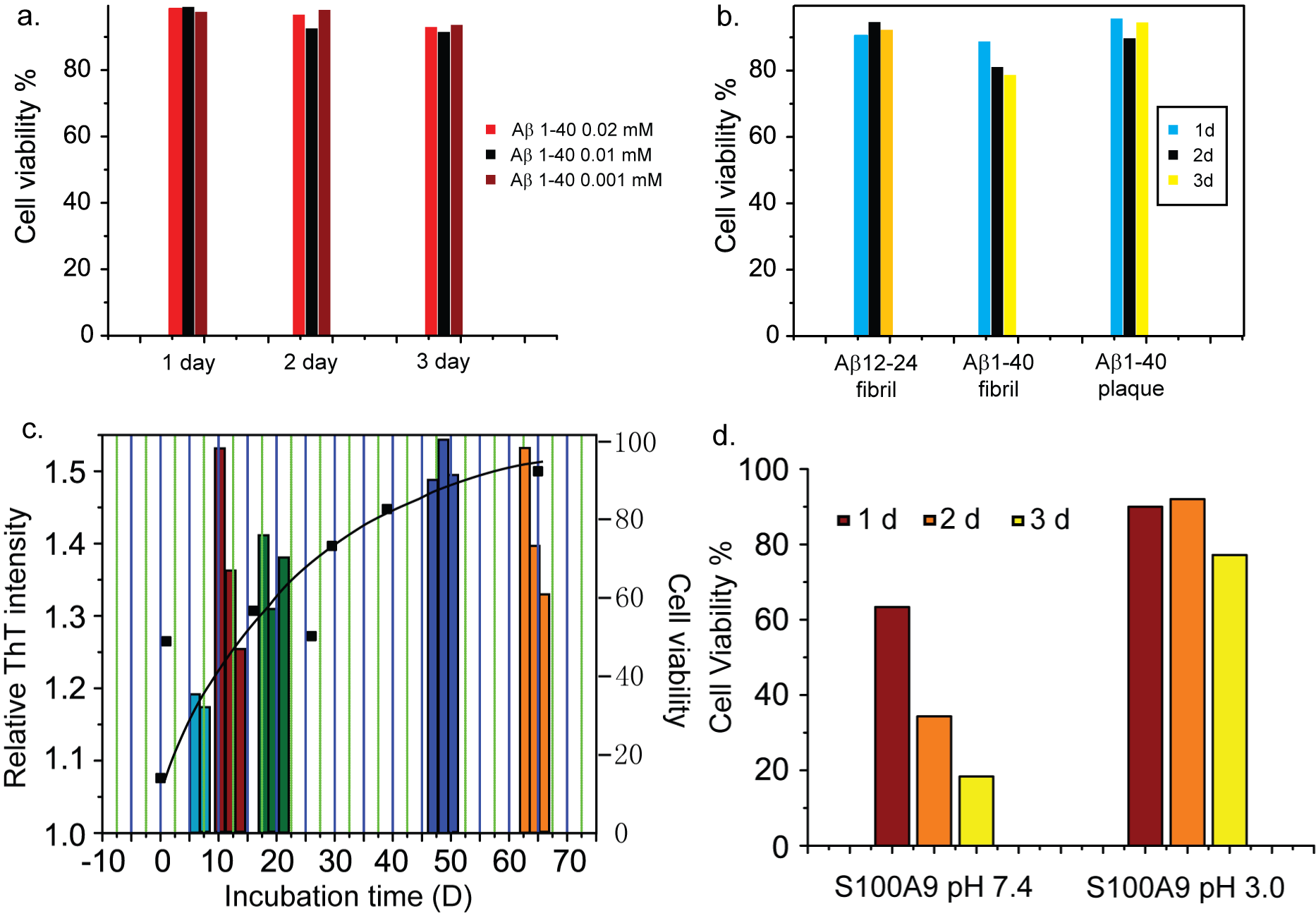

Supplement: Figure S4 — Measurements of NHSY5Y cell line viability by WST1 assay in the presence of (a): freshly dissolved A1–40 at pH 7.4. Different colors denote different concentrations of A1–40. (b): 0.2 mM A12–24 fibrils, 0.2 mM A1–40 fibrils and 0.2 mM A1–40 plaque. A1–40 plaques were formed by incubation at pH 3.0 and 37C for 3 d. To avoid pH effect, pre-formed A plaques were carefully dialyzed to pH 7.4. A12–24 and A1–40 fibrils were formed by incubation at pH 7.4 and 37C for over 10 d. (c) The relationship between the kinetics of S100A9 amyloid formation (Relative ThT: left Y axis) at pH 3.0 and the effects of S100A9 (0.02 mM) on NHSY5Y cell viability (WST1 assay: right Y axis). Before mixing with cells, S100A9 was incubated at pH 3.0 and 4C for up to 70 d. Aliquots were taken for measurements of ThT and cytotoxicity at different time during incubation. The columns in each group correspond to 1 to 3 d of incubation with cells (In day 6 aliquots, S100A9 samples were incubated with cell for up to 2 d). (d) Measurements of NHSY5Y cell line viability by WST1 assay in the presence of S100A9 (0.02 mM) at pH 7.4 and 3.0. Different colors denote 1 d to 3 d of incubation with cells. (TIF) [file pone.0032953.s004.tif]

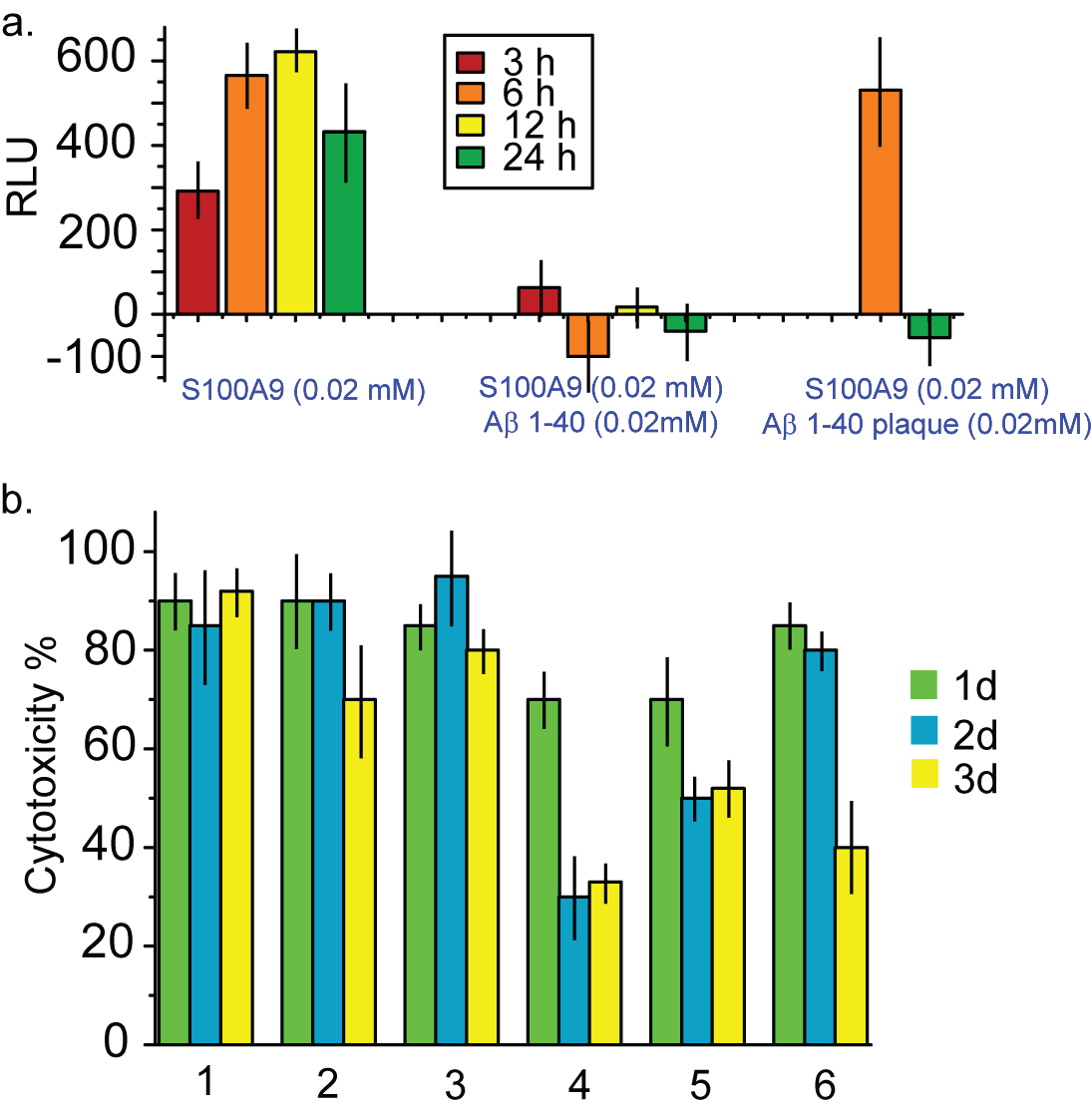

Supplement: Figure S5 — (a) Luminescent cytotoxicity assay of neuron cells in presence of 0.02 mM S100A9 and S100A9/A1–40 mixture. S100A9 and the mixture were incubated at 37C for 3, 6, 12 and 24 h before mixing with cells. A1–40 plaques were formed by incubation at pH 3.0 and 37C for 3 d. To avoid pH effect, pre-formed A plaques were carefully dialyzed to pH 7.4 before mixing with S100A9. Utilizing cortical neuron cells, the CytoTox-Glo Cytotoxicity assay uses a luminogenic peptide substrate, the AAF-Glo, to measure the activity of dead-cell protease, which is released from cells that have lost membrane integrity. The AAF-Glo substrate cannot cross intact cell membranes and does not generate any appreciable signal from the live-cell population. (b) Measurements of NHSY5Y cell line viability by WST1 assay in the presence of freshly dissolved (1) 0.2 mM A12–24; (2) 0.2 mM A12–24 and 0.02 mM S100A9 mixture; (3) 0.2 mM A1–16; (4) 0.2 mM A1–16 and 0.02 mM S100A9 mixture; (5) 0.2 mM A1–42; (6) 0.2 mM A1–42 and 0.02 mM S100A9 mixture. (TIF) [file pone.0032953.s005.tif]

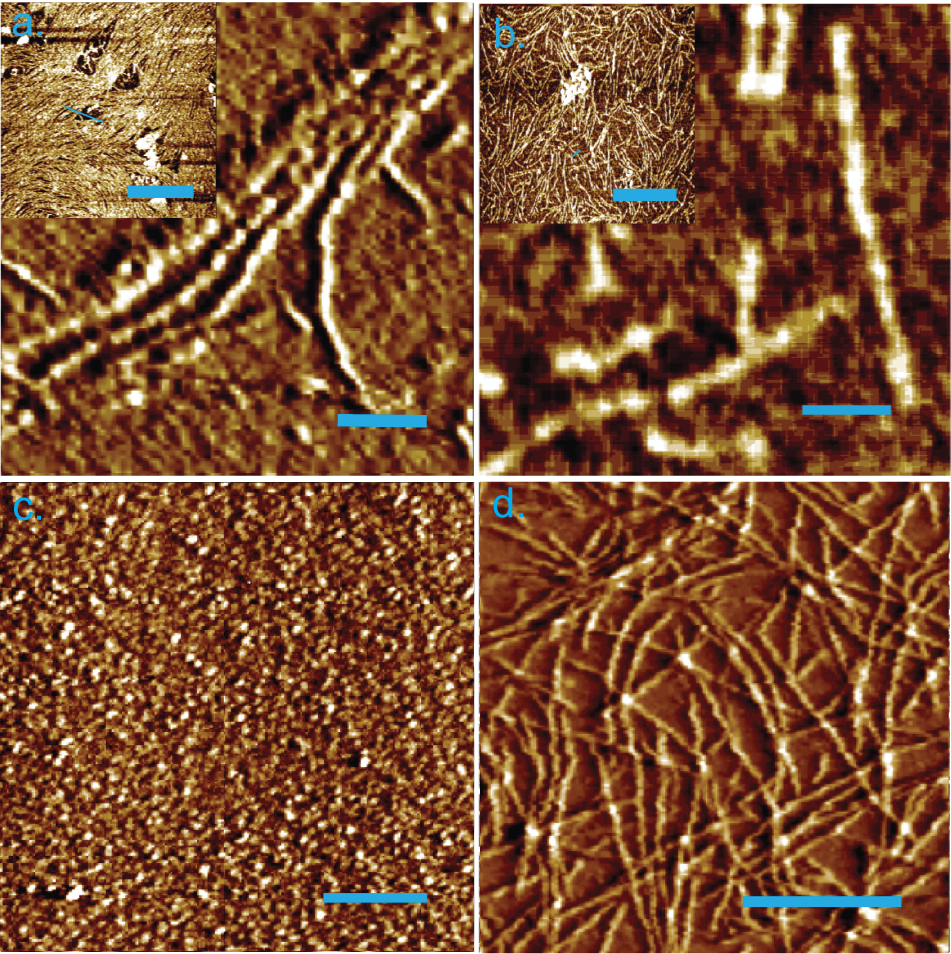

Supplement: Figure S6 — AFM images of (a) A12–24 peptide (0.2 mM) incubated at pH 7.4 and 37C for 3 d; (b) the mixture of freshly dissolved A12–24 peptide (0.2 mM) and S100A9 protein (0.02 mM) incubated at pH 7.4 and 37C for 3 d; (c) A1–42 peptide (0.2 mM) incubated at pH 7.4 and 37C for 3 d; (d) the mixture of freshly dissolved A1–42 peptide (0.2 mM) and S100A9 protein (0.02 mM) incubated at pH 7.4 and 37C for 3 d. In fig. a and b, the scale bars denote 100 nm in the figure, and 1000 nm in the insertion, respectively. The scale bars denote 200 nm in fig. c and 500 nm in fig. d. With the addition of S100A9, large quantities of A12–24 amyloid fibrils (2 nm in height) were formed, which is similar in size with the mature A fibrils. In contrast, only protofibrils (0.5 nm in height) were observed with A12–24 control samples under the same conditions (panel a). Similarly, A fibrils were only formed in the presence of S100A9 after 3 d incubation at pH 7.4 and 37C. The average height of the fibrils in fig. d is 2 nm, which is close to the reported value for A amyloid fibrils. (TIF) [file pone.0032953.s006.tif]
